# The emergence of psychoanalytical electrochemistry: the translation of MDD biomarker discovery to diagnosis with electrochemical sensing

**DOI:** 10.1038/s41398-022-02138-y

**Published:** 2022-09-08

**Authors:** Priyanka M. Nadar, Mckenna A. Merrill, Katherine Austin, Stephen M. Strakowski, Jeffrey M. Halpern

**Affiliations:** 1 Department of Chemical Engineering, University of New Hampshire, Durham, NH 03824 USA; 2 College of Medicine, Drexel University, Philadelphia, PA USA; 3 Department of Psychiatry, Dell Medical School, University of Texas at Austin, Austin, TX USA

**Keywords:** Diagnostic markers, Psychiatric disorders

## Abstract

The disease burden and healthcare costs of psychiatric diseases along with the pursuit to understand their underlying biochemical mechanisms have led to psychiatric biomarker investigations. Current advances in evaluating candidate biomarkers for psychiatric diseases, such as major depressive disorder (MDD), focus on determining a specific biomarker signature or profile. The origins of candidate biomarkers are heterogenous, ranging from genomics, proteomics, and metabolomics, while incorporating associations with clinical characterization. Prior to clinical use, candidate biomarkers must be validated by large multi-site clinical studies, which can be used to determine the ideal MDD biomarker signature. Therefore, identifying valid biomarkers has been challenging, suggesting the need for alternative approaches. Following validation studies, new technology must be employed to transition from biomarker discovery to diagnostic biomolecular profiling. Current technologies used in discovery and validation, such as mass spectroscopy, are currently limited to clinical research due to the cost or complexity of equipment, sample preparation, or measurement analysis. Thus, other technologies such as electrochemical detection must be considered for point-of-care (POC) testing with the needed characteristics for physicians’ offices. This review evaluates the advantages of using electrochemical sensing as a primary diagnostic platform due to its rapidity, accuracy, low cost, biomolecular detection diversity, multiplexed capacity, and instrument flexibility. We evaluate the capabilities of electrochemical methods in evaluating current candidate MDD biomarkers, individually and through multiplexed sensing, for promising applications in detecting MDD biosignatures in the POC setting.

## Introduction

Major depressive disorder (MDD) is a common psychiatric disease defined as a persistently low mood and anhedonia for 2 weeks or more [1]. Diagnosis requires the presence of four or more of the symptoms alongside depressed mood/anhedonia that include sleep disturbances, feelings of worthlessness or guilt, low energy, poor concentration, appetite changes, psychomotor changes, and suicidal ideation [1]. The impact of depression is significant, with over 264 million people worldwide affected, making it the leading cause of disability [2]. Despite these ramifications, many people fail to receive effective treatment as they are not correctly diagnosed.

The standard for psychiatric diagnosis in the United States is the fifth edition of the Diagnostic and Statistical Manual of Mental Disorders (DSM-V), which provides evidence-based criteria and guidelines for diagnosis. Physicians are encouraged to also use clinical acumen when evaluating both the severity and etiology of psychiatric symptoms. Consequently, a psychiatrist’s medical opinion may be affected by their own personal biases as well as the cultural lens through which their clinical training occurred. Moreover, people with depression often describe symptoms in ways that are hard to align with standardized criteria sets. Depression encompasses multiple types of symptoms and occurs on a spectrum of severity, which further complicates diagnoses. These characteristics of depression reaffirm the need for objective metrics of physiological disruption in the depressed state.

In medicine, biomarkers serve as a bridge between molecular dysregulation, clinical presentation, diagnosis, and treatment response. By understanding the interplay among these factors, clinicians can anticipate the natural history of a person’s disease, as well as response to treatment and potential long-term consequences. Robustly validated biomarkers are routinely monitored to chart disease evolution and progression in many conditions, including troponin/CK-MB for heart disease, ALT/AST for liver disease, and BUN/cystatin C/creatinine for kidney disease [3, 4]. The markers for the aforementioned diseases are sensitive to the damaged organ, but not specific to a singular etiology; specificity is granted by the integration of patient history and presenting symptoms [3, 4]. This approach is possible due to a clearer understanding of the pathogenesis of these diseases; however, it still relies on synthesizing information from multiple diagnostic sources and molecular pathways. With the heterogeneity of psychiatric presentations, even within a DSM category, other well-validated chemical markers would aid diagnosis and treatment. This approach is especially important as recent studies suggest that we must expand our pathophysiological understanding of depression beyond the serotonin model [5].

An emerging interest in the study of biomarkers and biochemical pathways indicative of various mental diseases is gaining traction in both biochemical and psychological communities to address this need for objectivity. As with the clinical management of these conditions, biomarkers for depression could play an important role in the diagnosis and treatment of depression. Judiciously collected biomarker data have three potential applications: (1) an integrated understanding of its physiological effects of depression on the human body; (2) development of accessible and more precise diagnostic, prognostic, and therapeutic modalities; and (3) innovation in passive monitoring of people with depression. Passive monitoring could prove to be especially useful for people with depression as active efforts to track their mental health may be difficult to sustain. Any practical diagnostic modality must effectively compile data from multiple sources in order to create a standardized panel.

The development of a panel-based biomarker profile for MDD may be very helpful for improving diagnostic and therapeutic outcomes. To this end, a robust and accurate molecular detection platform is needed for the eventual translation of the biomarker panel to point-of-care (POC) settings. Here, we report on various molecular pathways disrupted in major depression and identify several biomarkers from these pathways that provide examples as to what molecular subtypes might be identified and integrated into a feasible biomarker panel. Moreover, we evaluate the advantages and disadvantages of technologies commonly used in biomarker selection for their applications to clinical translation. Modalities currently used for biomarker discovery are typically not suitable for POC settings and/or cannot achieve the necessary sensing platform for MDD’s heterogeneous biomarker profile. We propose that electrochemical sensing platforms offer promise for diverse molecular detection, multiplexed capacity, and device suitability.

## Biomarkers and precision medicine

Currently, major depression is clinically subdivided into melancholic, atypical, and anxious subtypes based on the presence of specific symptoms. However, treatment response is not necessarily aligned with these subtypes, and it is not clear if they are maintained within an individual over time. To address this discrepancy, several molecular models for the pathogenesis of depression have been proposed. Animal models and preliminary clinical studies have expanded our understanding of depression from merely an imbalance in serotonin and other monoamines in the central nervous symptoms to include neuroimmune modulation, interruption of the hypothalamic–pituitary–adrenal (HPA) axis, and growth factor dysregulation may also play an important role in depression and its sequelae [6].

Several efforts to understand these mechanisms of disruption are underway. Most efforts to identify biomarkers in depression provide inconclusive results when subjected to clinical trials and robust meta-analyses. This observation may be due to a lack of standardization in experimental and analytical methods. The i-SPOT-D trial attempts to rectify this problem by providing a standardized framework to guide the analysis of biomarker data from imaging, chemical, genetic, and psychological studies and correlate them with treatment response to common anti-depressants [7]. However, solid conclusions arising from these data relevant to POC technology have yet to be drawn.

As the healthcare field continues to implement evidence-based medicine into clinical decision making, there is a need to collect more quantitative data on the physiological status of affected people as compared to unaffected comparison subjects. Currently, several biomarkers have been identified as having potential importance in the pathogenesis and clinical presentation of depression (Table 1). Biomarker levels and other physiological signals on their own cannot provide the full picture of disease manifestation, but they may be a crucial addition to providing a clearer picture of the individual’s condition alongside their symptoms, behaviors, and medical history. We have chosen to evaluate chemical biomarker samples found in peripheral blood, saliva, and urine for ease of detection in a POC application. These molecules, while not necessarily definitive markers that could be used in a diagnostic screening panel for depression, highlight the diversity of dysregulated pathways and molecule types that could be monitored for an integrated picture of the manifestation of depression in an individual.

**Table 1:** Potential candidate MDD biomarkers.

Candidate	Pathway	Function	Specimen	Analysis	Reference
8-OHdG	Oxidative stress	Marker of DNA damage	Blood, urine	ELISA, competitive immunoassays	[100]
Apolipoprotein D	Oxidative stress	Lipid transport protein found in the brain and testes	Blood	LC-MS	[13]
Apolipoprotein B	Oxidative stress	Systemic lipid transport protein	Blood	LC-MS	[13]
Vitamin D-binding protein	Oxidative stress	Vitamin D metabolite transport	Blood	LC-MS	[13]
Ceruloplasmin	Oxidative stress	Copper transport	Blood	LC-MS	[13]
Hornerin	Oxidative stress	Role in depression unclear	Blood	LC-MS	[13]
Profilin 1	Oxidative stress	Actin-binding protein	Blood	LC-MS	[13]
Kynurenine	Niacin production	Tryptophan metabolite	Blood, urine	GC-MS	[14]
Quinolinic acid	Niacin production	Toxic metabolite of kynurenine	Blood, urine	HPLC	[15]
GABA	Neurotransmitter	Inhibitory neurotransmitter	Blood	GC-MS	[14]
Tyramine	Catecholamine release	Tyrosine derivative	Blood	GC-MS	[14]
Dopamine	Neurotransmitter	Excitatory neurotransmitter	Blood	GC-MS	[14]
BDNF	Neurotrophic factor	Protein that supports neural growth and differentiation and normal neuronal function	Blood	ELISA	[19]
miRNA-132	Neuroinflammation modulator	Neural signaling	Blood	RT-PCR	[22]

### Inflammation and oxidative stress

Many of the most robustly validated circulating molecular biomarkers implicated in depression are cytokines involved in the pathway for general inflammation (e.g., IL-6, TNF-α, C-reactive protein (CRP), and interferons) [8]. Levels of these compounds are elevated in many different illnesses, including cancer, heart disease, sepsis, and infection. However, depression is a peculiar state in which both the “stress hormone” cortisol and inflammatory markers are increased, even though cortisol is well-known as an anti-inflammatory agent. This phenomenon may be due to glucocorticoid receptor resistance interrupting the HPA axis. Without feedback inhibition, cortisol is excessively secreted, but the inflammatory response remains activated. Consequently, there are two dysregulated components that may have an effect on mood: high cortisol and high levels of inflammatory cytokines. Elevated levels of cortisol have long been correlated with depressive symptoms; one study measures these inflammatory cytokines, cortisol, and nesfatin-1 (an anorexigenic peptide) and finds that they can predict the depressive state with 97% specificity [9]. Outside of the HPA axis, inflammation also appears to significantly impact other neuro-regulatory systems, such as the serotonin, dopamine, and glutamate pathways. Inflammation is also an activator of the kynurenine pathway, which produces the toxic metabolite quinolinic acid [8]. Finally, inflammatory molecules create a “sick state” (e.g., the flu) that mimics many of the symptoms of depression, including anhedonia, fatigue, low mood, loss of appetite, and sleep disruption.

One potential trigger of the chronic inflammation pathway is an increase in free radical injury via oxidative stress. A 2015 meta-analysis found that biomarkers for oxidative stress, such as 8-hydroxy-2’-deoxyguanosine (8-OHdG) and F_2_-isoprostanes were often elevated in the urine and peripheral blood of people diagnosed with depression, especially women [10]. A more recent systematic review validated 8-OhdG and F2-isoprostanes as a biomarker of depression and added 3-NT, PC, 4-HNE, 8-isopostrane (8-Iso), malondialdehyde (MDA), SOD, CAT, GPx, and vitamins A and C [11]. 8-OhdG is a marker for DNA damage and F_2_-isoprostanes are eicosanoids that serve as markers for lipid damage. However, oxidative stress markers are also elevated alongside general inflammatory markers in people with cardiovascular disease and diabetes, which interferes with their ability to be used alone as markers of depression [12].

Along with direct markers of oxidative stress, the presence of protective factors can potentially be used as a marker of depression. A 2016 study isolated a panel of 6 peripheral blood biomarkers that differentiated between drug-naive depressed and healthy women with 67% sensitivity, 69% specificity, and 68% overall classification accuracy: apolipoprotein D, apolipoprotein B, vitamin D-binding protein, ceruloplasmin, hornerin, and profilin 1 [13]. These proteins are involved in protecting the central nervous system from oxidative injury from the inflammatory state that depression mediates as well as transport of key functional cofactors like vitamin D and copper throughout the body.

### Neuroactive compounds and their metabolites

Measuring neuroactive compounds and their metabolites may be the most obvious choice for monitoring the pathogenesis and disease course of depression. A potential biomarker panel of kynurenine, GABA, tyramine, and dopamine was proposed in a 2018 metabolomics study on neurochemical changes specific to the early stages of depression, allowing physiological differentiation between unipolar major depression and bipolar disorder [14]. GABA and dopamine are both neurotransmitters, while kynurenine (a derivative of tryptophan that may be related to abnormal serotonin production) and its metabolites are important in the neural remodeling. Neurotoxic kynurenine metabolites such as 3-hydroxykynurenine and quinolinic may play a role in reducing cortical thickness in depressed people [15]. Generally, kynurenine levels have been shown to be reduced in depressed people while levels of its metabolite quinolinic acid are increased; kynurenine is detectable in peripheral blood and quinolinic acid is detectable in urine [16].

### Genetic variants

Several potential genetic candidates have been isolated to potentially play a role in the pathogenesis of depression. Detecting changes in the expression of target genes may help stratify different phenotypes of MDD as well as predict response to both pharmacological and psychological interventions [17]. One example is a brain-derived neurotrophic factor (BDNF), a growth factor shown to be lowered in depression, and BDNF can be used as a biomarker for neuropsychiatric illness [18]. However, specific variants in protein structure may also play a role in the pathogenesis of depression and provide more specificity for depression. A meta-analysis review has shown that low BDNF is a promising marker for the presence of depression and response to treatment [19]. Single-nucleotide polymorphic (SNP) variants of BDNF, such as rs6265 (Val66Met), increase the binding ability of the 5-HT_1A_ receptor, a potential endophenotype of depression [20]. Detection of key BDNF variants via genomic analysis of rs6265 in peripheral blood samples has shown to be more specific in identifying depression in certain populations [21]. The rs6265 allele has also been implicated in susceptibility to depression resulting from different types of life stressors. While the evidence for rs6265 is mixed, it can be considered an example of potential variants to use for screening.

Peripheral mRNA mRNAs and miRNA molecules have also been shown to be dysregulated in depression. They are detectable in peripheral blood and several potential candidates may serve as biomarkers for diagnosis and for evaluating pharmacological treatment [22]. For example, miRNA-132 plays a role in a multitude of pathways in the brain [23–25] and is proposed to be a regulator of neuroinflammation by altering the expression of BDNF [22]. A systematic review showed miR-24-3p, let 7a-5p, miR-26a-5p, miR135a, miR-425-3p, miR-132, miR-124, and miR-16-5p to be circular miRNAs with the most evidence behind their correlation to MDD, but a definitive conclusion could not be drawn as to which molecules are best for use as a biomarker [26]. Several functionally related miRNAs are carried in exosomal vesicles, an avenue that has shown promise in tracking other neurological and psychological disorders, but which needs more characterization in depression.

### Panel-based biomarker detection

As evident from the other biomarkers discussed, the molecular pathogenesis of depression appears to be heterogeneous and complex, with many intersecting pathways. Therefore, a panel of metabolites and the abovementioned biomarkers, rather than a single marker or subtype of marker, may be the most optimal way of creating a diagnostic test. For example, in one study, urinary detection of the metabolites N-methylnicotinamide, aminomalonic acid, azelaic acid, and hippuric acid was used to create a panel specific to depression. The area under the ROC was found to be 0.977 in the training set and 0.934 in the testing set, indicating that this panel robustly separates depressed and non-depressed people [27]. These metabolites span various dysregulated pathways within the body, such as impaired fatty acid oxidation, the gut microbiome, and neurotransmitter synthesis. Other panels use a target to survey immune and neuroendocrine disruption; a panel of 33 target molecules was identified by performing a meta-analysis of other studies looking into blood-based inflammatory changes in depressed individuals [28]. A panel-based approach is also key to integrating microbiome data, which may be of growing relevance to our understanding of psychiatric dysregulation [29]. Sampling across various metabolic pathways can potentially differentiate among types of depression and predict treatment response. Other panels have included clinical imaging and socioeconomic parameters alongside proteomic data to screen for subthreshold depression and moderate predictive ability for escalating to MDD, providing a pathway by which biomarker data are integrated into a multi-faceted, panel-based approach [30, 31]. At this point, the evidence for any individual biomarker is not robust; a panel-based approach may solve issues of validity and specificity to depression.

### Current point-of-care technologies for diagnosis

**Fig. 1:**
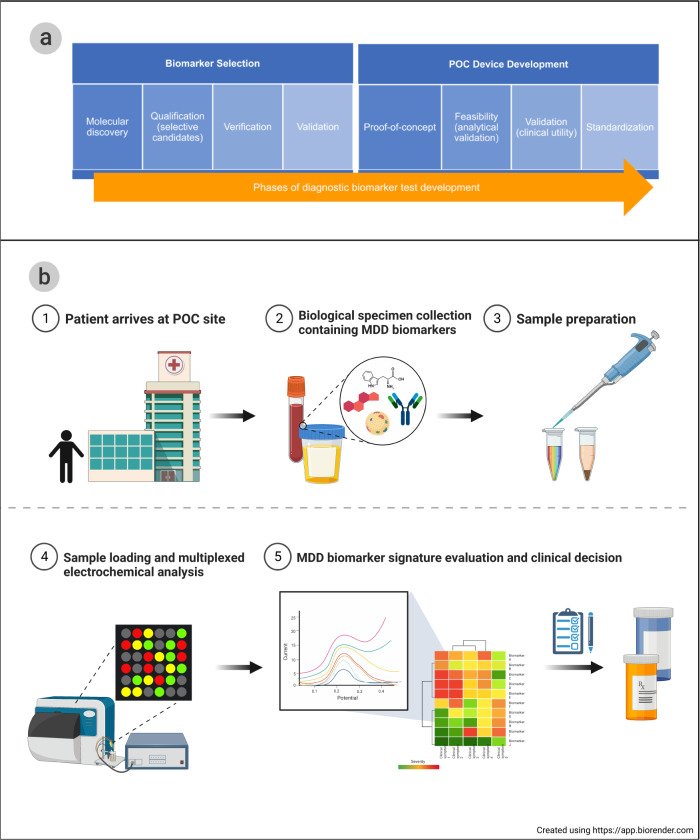
Lifecycle and application of a point-of-care diagnostic device. **a** Process of developing a POC diagnostic device and **b** flow diagram of utilizing a POC diagnostic device for clinical decision making. The figure was created with BioRender.com.

We propose that a panel-based POC diagnostic test for MDD is likely the most optimal method to address both research and clinical needs. POC diagnostic tests aim to provide more streamlined clinical decision making in personalized treatment and pharmacodynamic drug monitoring, ideally leading to faster assessment of treatment interventions. Technologies used for POC must undergo the phases of development found in Fig. 1a prior to clinical application.

Current methods used for depression candidate biomarker discovery (e.g., LC-MS, enzyme-linked immunosorbent assay (ELISA)), provided in Table 1, offer high throughput and untargeted approaches in distinguishing over 1000 analytes for biomarker profile analysis. However, LC-MS or ELISA are typically too complex for POC settings (i.e., clinics and center laboratories) for depression diagnostic analysis. As an alternative, electrochemical sensing platforms have favorable characteristics for POC diagnostic applications and panel biomarker profile testing for depression.

The ELISA is the most commercially available approach of a potential POC device. ELISA provides various platforms for analyte detection including direct, indirect, sandwich, and competitive binding with multiplexed capabilities. ELISA platforms are limited to detecting analytes with available selective ligands or antibodies. In addition, ELISAs often call for a label to detect a target for analyte detection, which can increase time, expense, and sample handling.

Commercial DNA-based assays utilize polymerase chain reaction (PCR) for POC diagnostic testing. As shown in Table 1, PCR is used to detect various DNA derivatives, such as DNA, plasmid and genomic RNA, mRNA, and miRNA [25]. qPCRs provide high sensitivity and short reaction times of 30–55 min, thus providing a desirable diagnostic instrument. Highly compact, miniPCRs are becoming more commercially available as cost-effective and portable alternatives [32, 33]. RT-PCR and RT-qPCR have revolutionized viral disease POC testing through viral DNA sequence amplification, such as for Ebola, Zika, and the most recent SARS-CoV-2 [33, 34]. The major limitation to RT-PCR, however, is the technology is constricted to detecting and quantifying genetic/genomic information in a biological specimen, which prevents its application to biomarker panel analyses that include circulating proteins, metabolites, and macromolecules.

Chromatography and mass spectroscopy technologies provide the necessary characteristics for biomarker panels, including high sensitivity, multiplexing of thousands of analytes with extended compound range, high sample throughput, and microsample volume requirements [35–37]. Miniaturizing HPLC systems has become a growing interest, resulting in technologies such as μHPLC and nHPLC. However, diagnostic applications of HPLC and LC-MS methods are limited due to the initial costs of permanent equipment, long-term instrument performance maintenance, and costs of personnel with the requisite expertise, which prohibit its implementation in smaller laboratories and clinics. LC-MS requires complex sample preparation and instrument operation as well as intricate data interpretation, and thus poses issues in standardizing the overall operation for multi-laboratory practices. Limitations of HPLC and LC-MS techniques prohibit the 24/7/365 availability of the platform required for clinical settings such as psychiatric facilities and applications like therapeutic drug monitoring [38].

## Suggested electrochemical point-of-care approaches

The methods previously discussed typically require trained analytical scientists, and therefore, are not viable POC testing during a physician visit or at-home monitoring. POC settings require diagnostic technologies to be non-invasive, quantitative, specific, sensitive, and low-cost with rapid throughput and turnaround time, standardized data interpretation, and minimal training requirements [39]. Additional characteristics are required for resource-limited clinics and settings (e.g., patients’ homes). Therefore, we propose electrochemical sensing platforms as a way to build panel-based assays that can be more broadly used. In order to deliver a clinical utility to major depression evaluation, a POC technology will additionally evaluate biomarker species of different subtypes (i.e., proteomic, metabolomic, transcriptomic, epigenetic, genetic) for panel inclusion. Here, we examine current electrochemical sensing platforms developed to be applied toward the detection of biomarkers identified to metabolic pathways commonly disrupted in MDD. Electrochemical sensing platforms currently exist, and some have been validated for clinical applications.

Electrochemical sensing platforms are characterized by high sensitivity, portability, ease of use, simple data interpretation, cost-effectiveness, reduced sample volume, precision, accuracy, short analysis time, and multiplexed capabilities [40–43]. Electrochemical-based devices can be integrated into miniaturized microfluid systems to enhance portability, automate sample preparation, and reduce non-specific binding and crosstalk in signal readout. Miniaturized electrochemical sensors and Lab-on-a-Chip technologies remove bulk equipment requirements and reduce instrument costs, making them more suitable devices for smaller labs and clinics [44]; smaller dimensions allow for smaller sample volumes and reduce reagent requirements needed for analysis. Electrochemical sensing utilizes both labeled technology and label-free technology, thus expanding its detection diversity compared to ELISA. Label-free detection enables real-time target-probe binding, increases the speed of detection by removing labeling steps, and promotes device simplicity. Electrochemical systems can also detect analytes that are both electrochemically and non-electrochemically active [45]. Electrochemical systems also sense a variety of clinically relevant biomolecules, ranging from small molecules to nucleic acids and proteins in a wide range of biological samples, such as urine, blood, CSF, and saliva [46]. Recent developments in cross-reactive biosensors also introduce the ability to detect volatile molecules for biomarker panel analysis [47–49]. Biomarker selectivity and instrument readiness is discussed.

### Protein detection

Like other immunoassay techniques, electrochemical detection of inflammatory markers, like IL-6 and TNF-α, primarily utilizes antibodies for sensitivity and selectivity. Unlike ELISA platforms, electrochemical sensing systems can sensitively detect IL-6 with label-free strategies [50–53]. The fundamental benefit of label-free techniques is the elimination of additional washing steps required for target-label interaction, thereby reducing sample preparation complexity and time. Moreover, removing the need for labeling can also reduce complexity and thereby training of personnel, cost, and time among other factors. One label-free sensing platform utilized IL-6R immobilized on single-walled carbon nanotubes (SWCNTs) to promote electron-transfer reactions and increase electrode surface area, thereby enhancing sensor sensitivity [54]. The sensor’s specificity was confirmed against interfering compounds commonly found in biological fluids (bovine serum albumin, cysteine) [54].

Label-free strategies for TNF-α have been achieved using electrochemical sensing platforms. In one case, a TNF-α sensor was constructed with fullerene-functionalized carbon nanotubes and ionic liquid (C_60_-CNT-IL) and TNF-α antibody entrapment for the sensitive, label-free detection of TNF-α in serum. A miniaturized and low-cost sensing device was also used to determine this compound [55]. Sensing and recognition elements were created by the immobilization of CMA and TNF antibodies on gold electrodes [55]. The device was capable of performing 8 parallel measurements for either single measurement redundancy or multiplexed detection purposes. The miniaturized device exhibits promising qualities for POC applications; however, physiologically relevant sensitivity of detection must be achieved using biological samples. The most recent work involving TNF-α electrochemical sensors has shown great sensitivity improvement. However, other parameters still leave much to be desired as newer designs lack cost-effectiveness and require complex fabrication processes [56].

Electrochemical mechanisms have been developed for other potential MDD biomarker such as CRP. Two sandwich-type electrochemical immunoassays have been developed for serum CRP detection [57, 58]. A CRP-selective sandwich immunosensor was fabricated using copper nanoparticles as a signaling molecule coupled with a hybridization chain reaction to amplify the resultant signal [58]. The copper-based sensor achieved proof-of-concept testing for sensitive and selective detection of CRP in the presence of interfering compounds (AFP, CEA, L-Cys, lysine, uric acid) [58]. The performance of the sensor was validated via recovery of spiked clinical samples. Results were confirmed by an immunofluorescence assay reference method [58]. A different sensor fabrication strategy utilized anthraquinone-labeled secondary antibodies on dual screen-printed gold electrodes (SPGE) for simultaneous CRP detection and negative control confirmation [57]. Analytical validation of the SPGE immunosensor was performed by CRP human serum sample assay. Again, concentrations were certified by a standard method [57]. Serum sample recovery and negative control confirmation were in acceptable statistical agreement with the certified standards [57].

Another potential biomarker, BDNF, can be detected electrochemically through the immobilization of anti-BDNF monoclonal antibodies [59, 60]. For example, a POC electrochemical immunosensing device, known as EndoChip, was fabricated for the detection of circulating BDNF. Results from the EndoChip were in good correlation to ELISA results using endogenous samples with less sample preparation and more rapid analysis [60]. The investigation of SNP variants in depression phenotypes is a burgeoning field, thus electrochemical systems have been designed to identify and distinguish a range of single-nucleotide polymorphisms [61]. Through the use of single-stranded DNA probes designed for each target SNP, multiplexed SNP detection can be expanded to incorporate other desired SNPs of interest, potentially aiding in the distinction between BDNF variants. A separate CMOS-based Lab-on-a-Chip platform was used for SNP determination [62].

For certain proteins, detection technologies may not exist; however, structurally relevant analogs that have been electrochemically detected demonstrate similar platforms. For example, Vitamin D-binding protein is known to be structurally homologous to albumin, which has been electrochemically detected using molecularly imprinted polymers and a redox probe analyte [63]. Similarly, ApoD and ApoB are the main components of high-density lipoproteins (HDLs) and low-density lipoproteins (LDLs), respectively. LDLs have been detected through electrochemical systems, along with its component, ApoB-100 [64, 65]. HDLs have also been detected. HDL and LDL detection depends on binding with antibodies specific to components of the lipoprotein, being anti-ApoB-100 for LDL and Anti-ApoA1 for HDL [64, 65]. The lack of electrochemical detection of ApoD may be due to the lack of ApoD-specific antibodies as well as the lack of incentive to develop a biosensor for its detection.

### Amino acid and neurotransmitter detection

Many neurotransmitters are derivatives of amino acids and exist in trace amounts; molecular detection of neurotransmitters requires sensitivity and specificity in order to differentiate between structurally similar derivatives in small quantities [66, 67]. Biosensors have been fabricated for the quantification of kynurenine metabolites in trace amounts within biological samples. For example, a biosensor was fabricated based on the enzymatic interaction of quinolinate phosphoribosyl transferase for the determination of trace quinolinic acid in serum samples, which was more sensitive compared to HPLC-ECD methods [68].

Due to their natural electroactivity, most neuroactive compounds can be detected simultaneously using multiplexed systems [69–71]. Electrochemical sensors can utilize the surfactant sodium dodecyl sulfate to improve the detection of dopamine and serotonin by creating surface charge effects to electrostatically promote sensitivity [72]. Use of carbon materials or fast-scan cyclic voltammetry has been shown to improve sensitivity toward and differentiation between dopamine and serotonin [73–75]. The design of dopamine-selective biosensors has integrated materials such as screen-printed electrodes, graphene-modified microfluidic paper-based analytical devices, and pencil-on-paper analytical devices to improve device cost and simplicity [72, 76]. Other neurotransmitters, like GABA and acetylcholine, are not electrochemically active; detection platforms often rely on converting non-electroactive compounds to redox-active components by derivatization agents or utilizing ITIES pipet electrodes [77]. In addition, paper-based biosensors for detecting neurotransmitters and other neuroactive compounds from blood offer low-cost, user-friendly POC testing [78].

Base amino acids can also be monitored electrochemically in the blood. New techniques are using metal oxide derivatives and carbon nanostructured surfaces to electrocatalytically activate amino acid structures [79–81]. Recent advances have indicated that high sensitivity with some selectivity can exist in determining neurotransmitters based on measuring blood amino acid metabolite derivatives [79]. However, many of these sensors also form Schiff base complexes with arginine, a common amino acid, reducing the overall stability of the sensor surface [82].

### Nucleoside detection

8-OhdG serum and urine levels are increased in MDD in response to antidepressant treatment. While ELISA kits provide selective detection of 8-OHdG via anti-8-oHdG monoclonal antibodies, electrochemical systems are also capable of sensitive and selective detection of 8-OHdG in biological samples [83, 84]. Electrochemical sensing of 8-OHdG can remove labeling requirements and incubation steps needed for ELISA techniques by utilizing the oxidation reaction of 8-OHdG. For example, a biosensor was constructed for the electrochemical determination of 8-OHdG in urine, blood, and serum samples using a paper-based electrode system [84]. The catalytic properties of the sensor were enhanced through carbon nanomaterial functionalization to improve 8-OHdG responses; by utilizing the oxidation potential of 8-OHdG, interfering signals from other electrochemically active species like ascorbic acid and uric acid were negligible [83, 84].

### Nucleic acid detection

Detection mechanisms for nucleic acids show promising advances due to their strong interactions with oligonucleotides . Multi-label multiplexed electrochemical systems have been used to selectively differentiate between miRNA sequences. Redox-labeled probes targeting RNA sequences are used to identify miRNA sequences for molecular detection [85]. A recent platform sensitively and specifically detected miRNA-182 and miRNA-381 with minimal interference from other miRNAs with similar sequences (miRNA-183, miRNA-300, miRNA-96, miRNA-382) [86]. Specificity was employed using oligonucleotide hairpin probes targeting miRNA sequences; the hairpin probes contained redox tags’ methylene blue and ferrocene for signal transduction of each miRNA [86]. Other redox tags that can be used in miRNA-labeled detection include oracet blue, as demonstrated in the individual detection of miRNA-155 [87]. Because specificity and selectivity are achieved by the sequence-specific targeting probe, this platform could potentially be expanded by additional hairpin probes specific to other miRNAs, such as miRNA-132. Furthermore, label-free electrochemical detection of miRNAs has also been developed [85, 88–90].

### Lipid detection

Compounds, such as lipids, are more difficult to sense compared to other potential biomarkers. F_2_-isoprostanes (F_2_-IsoPs) are prostaglandin F2*α*-like compounds that arise from the nonenzymatic oxidation of arachidonic acid and are classified into four different series based on chemical structure following oxidation. F_2_-IsoP quantification by GC-MS can take between 6 and 8 h, which is inappropriate for POC testing. 8-Iso has been detected electrochemically using a screen-printed electrode in serum samples with negligible interference from other biological compounds (e.g., adiponectin, BSA, cholesterol, ceruloplasmin, TNF-α, IL-6) [91]. Assay time was reduced to 1 h 30 min, significantly lower than those from GC-MS or ELISA techniques [91]. Assay results were validated using ELISA with RSD values below 2%, indicating high precision and excellent analytical agreement.

### Detection of other species

MDA is electrochemically detected most commonly from exhaled breath samples [92, 93]. One electrochemical sensor utilized the electro-oxidation of MDA from spiked human serum using a polytaurine film-modified gold electrode [94, 95]. A label-free sandwich electrochemical biosensor was constructed to capture MDA with human complement factor H using a sSWCNT-modified electrode backbone [96]. The biosensor was used to quantify free, bound, and total MDA levels in serum samples. Assay results for clinical samples were confirmed to provide higher detection sensitivity to MDA compared to UV-visible spectrophotometry, thus reinforcing the clinical utility of the proposed method.

### Device instrumentation for multiplexed sensing and POC application

Device instrumentation can be both expensive and too complex for integration in a POC setting; promising advances toward miniaturized and automated electrochemical systems have been explored. A group recently developed a miniaturized electrochemical device capable of eight parallel measurements, although the device has detected a single analyte for proof-of-concept purposes [55]. The miniaturized prototype significantly reduces the cost of permanent equipment to $300 compared to $3000–$100,000 for commercially available analyzers [55]. Similarly, the 256-sensor microfluidic array estimated a total cost of $200 [97]. The microfluidic pattern of the system can be easily modified to incorporate additional sensors, and the disposable nature of the device reduces its complexity [97]. A miniaturized 8-port manifold allows for uniform reagent loading for calibration purposes as well as for sample loading [97].

A significant number of fabricated sensors have reached the analytical validation stages of development and continue to progress toward clinical validation, clinical trials, and standardization. To provide clinical utility to biomolecular profiling for depression, a compact, multiplexed system must be constructed that integrates an array of individual sensors designated toward a collection of biomarkers. Multiplexed electrochemical systems offer simultaneous multi-analyte detection through the application of multi-electrode and multi-label platforms [98].

The main problem with developing a platform to detect multi-type biomarkers is the need for numerous sensing capabilities with diverse preparation requirements. Many individuals are trying to create new platforms that can achieve multi-type capabilities in electrochemical sensing. For example, a high-throughput electrochemical microfluidic array was designed to support the detection of 96 biomarkers in one sample with the potential to reduce turnaround time to under 60 min [97]. The prototype consisted of a 256-sensor system whose sensitivity and selectivity were confirmed for four biomarker proteins: PSA, IL-6, PF-4, and PSMA [97]. The sensing platform primarily used antibodies and magnetic nanoparticles as the fundamental sensing platform; however, there is potential for this array to integrate other sensing platforms for multi-type biomarkers. Another platform offers a promising advance toward multi-type biomarker detection in that both IL-8-mRNA and IL-8-protein were measured from saliva samples via a multi-electrode approach on a screen-printed electrode [99]; this multiplexed electrochemical system offered high sensitivity and selectivity to two heterogeneous inflammatory biomarkers with less sample preparation steps compared to PCR or ELISA [99]. The multiplexed capacity of this platform was limited, however, to two biomarkers due to the use of dual screen-printed electrodes. Other multiplexed platforms are presented in Table 2. As these systems incorporate additional sensors specific to individual analytes, the system’s complexity increases. Testing will need to be conducted for interference, device stability, and cross-reactivity. However, a multiplexed system tailored toward a standard MDD biomarker signature has yet to be achieved primarily because there is still an active debate on what a biomarker signature could look like. Despite this current uncertainty, it is evident that a robust, fast panel-based detection method would be impactful in the diagnosis and treatment of MDD.

**Table 2:** Examples of multiplexed detection of biomarkers relevant to MDD candidate biomarker panels by electrochemical systems.

Analyte	Electrochemical method	Multiplexed platform	Multiplexed capacity	Biospecimen tested	Reference
IL-1b, IL-10	CV, EIS	Multi-electrode, multi-label	8 working electrodes	Physiological medium	[42]
IL-8 mRNA, IL-8 protein	Amperometry	Multi-electrode	Dual screen-printed carbon electrodes	Saliva	[99]
PSA, PMSA, IL-6, PF-4	CV, DPV	Multi-electrode	Eight 32-sensor microfluidic immunoarrays connected via 8-port manifold	Serum	[97]
miRNA-182, miRNA-381	DPV, EIS	Multi-label	Multi-hairpin-ODN probes	Serum	[86]
Dopamine, norepinephrine	Electrochemical redox cycling	Multi-electrode	Microelectrode arrays	Artificial CSF	[71]
Kynurenine, tryptophan	DPV	–	BiF/BDDE	Culture medium	[70]
C-reactive protein, IL-6	CV, chronoamperometry	Multi-label	4 working electrodes	–	[101]

*CV* cyclic voltammetry, *EIS* electrochemical impedance spectroscopy, *WE* working electrode, *DPV* differential pulse voltammetry, *PSA* prostate-specific antigen, *PMSA* prostate-specific membrane antigen, *PF-4* platelet factor-4, *CA* constant-potential amperometry, *CNT* carbon nanotube, *BDDE* boron-doped diamond electrode, *BiF* bismuth film.

## Conclusions

Due to the physiological complexity and heterogeneity of major depression, POC testing will demand a multiplexed, multi-assay paradigm to distinguish a range of biomolecule types across metabolic pathways. As shown in Table 1, a range of molecular species is disrupted in depression phenotypes. Before MDD biomarker panels can be used in a diagnostic or treatment setting, two main events must occur: firstly, candidate biomarker panels must be validated in multi-site clinical trials with standardized technology, and secondly, biomarker panel tests must be approved for clinical settings following the stages illustrated in Fig. 1. Multiplexed electrochemical systems offer a promising direction for POC, diagnosis and therapeutic drug monitoring as user-friendly biosensing devices. Following validation and clinical acceptance of major depression candidate biomarkers, electrochemical sensor systems can be optimized for the detection of biomarker signature. In combining electrochemistry, electronics, and computer software, miniaturized multiplexed biosensing devices could offer a real-time diagnosis of greater accuracy than current purely clinical approaches, thereby reducing misdiagnoses and improving personalized treatment.

Expanding access to care and diagnostics is necessary to reduce the global disease burden of depression, which requires cheap and accessible resources and materials. Electrochemical sensing devices offer cheap methods for device fabrication that alleviate the cost and limitation associated with providing access to diagnostic care globally. Multiplexed electrochemical sensing is promising for diverse molecular detection, multiplexed capacity, and device suitability. However, electrochemical sensing devices are not developed sufficiently to fit these needs; multiplexed devices are in the early stages of evaluating the full extent of biomarker capacity. We recommend additional dedicated efforts toward developing sensing platforms for various molecular targets toward clinical utility. Most funds and effort are dedicated toward the new sensing paradigms or increasing sensor sensitivity instead of integration with robust instrumentation for the versatility of use. Once sensing strategies to detect a wide range of molecular targets are further developed, a diagnostic platform toward MDD could be proposed. However, we have explained strategies and potential MDD targets as sensing platforms are being developed.

## References

[1] Park LT, Zarate CA (2019). Depression in the primary care setting. N Engl J Med.

[2] James SL, Abate D, Abate KH, Abay SM, Abbafati C, Abbasi N (2018). Global, regional, and national incidence, prevalence, and years lived with disability for 354 diseases and injuries for 195 countries and territories, 1990–2017: a systematic analysis for the Global Burden of Disease Study 2017. Lancet.

[3] Thygesen K, Alpert JS, Jaffe AS, Chaitman BR, Bax JJ, Morrow DA (2018). Fourth universal definition of myocardial infarction (2018). J Am Coll Cardiol.

[4] Kwo PY, Cohen SM, Lim JK (2017). ACG clinical guideline: evaluation of abnormal liver chemistries. Am J Gastroenterol.

[5] Moncrieff J, Cooper RE, Stockmann T, Amendola S, Hengartner MP, Horowitz MA. The serotonin theory of depression: a systematic umbrella review of the evidence. Mol Psychiatry. 2022. 10.1038/s41380-022-01661-0. 10.1038/s41380-022-01661-0 PMC10618090 35854107

[6] Pariante CM (2017). Why are depressed patients inflamed? A reflection on 20 years of research on depression, glucocorticoid resistance and inflammation. Eur Neuropsychopharmacol.

[7] Williams LM, Rush AJ, Koslow SH, Wisniewski SR, Cooper NJ, Nemeroff CB (2011). International Study to Predict Optimized Treatment for Depression (iSPOT-D), a randomized clinical trial: rationale and protocol. Trials.

[8] Miller AH, Raison CL (2016). The role of inflammation in depression: from evolutionary imperative to modern treatment target. Nat Rev Immunol.

[9] Xu Y-Y, Ge J-F, Liang J, Cao Y, Shan F, Liu Y (2018). Nesfatin-1 and cortisol: potential novel diagnostic biomarkers in moderate and severe depressive disorder. Psychol Res Behav Manag.

[10] Black CN, Bot M, Scheffer PG, Cuijpers P, Penninx BWJH (2015). Is depression associated with increased oxidative stress? A systematic review and meta-analysis. Psychoneuroendocrinology.

[11] Barbosa ML, de Meneses A-APM, de Aguiar RPS, de Castro e Sousa JM, de Carvalho Melo Cavalcante AA, Sharbel Weidner M (2020). Oxidative stress, antioxidant defense and depressive disorders: a systematic review of biochemical and molecular markers. Neurol Psychiatry Brain Res.

[12] Black CN, Penninx BWJH, Bot M, Odegaard AO, Gross MD, Matthews KA (2016). Oxidative stress, anti-oxidants and the cross-sectional and longitudinal association with depressive symptoms: results from the CARDIA study. Transl Psychiatry.

[13] Lee MY, Kim EY, Kim SH, Cho K-C, Ha K, Kim KP (2016). Discovery of serum protein biomarkers in drug-free patients with major depressive disorder. Prog Neuro-Psychopharmacol Biol Psychiatry.

[14] Pan J-X, Xia J-J, Deng F-L, Liang W-W, Wu J, Yin B-M (2018). Diagnosis of major depressive disorder based on changes in multiple plasma neurotransmitters: a targeted metabolomics study. Transl Psychiatry.

[15] Meier TB, Drevets WC, Wurfel BE, Ford BN, Morris HM, Victor TA (2016). Relationship between neurotoxic kynurenine metabolites and reductions in right medial prefrontal cortical thickness in major depressive disorder. Brain Behav Immun.

[16] Ogyu K, Kubo K, Noda Y, Iwata Y, Tsugawa S, Omura Y (2018). Kynurenine pathway in depression: a systematic review and meta-analysis. Neurosci Biobehav Rev.

[17] Teperino R. Beyond our genes: the physiology of gene/environment interaction. Switzerland: Springer; 2020.

[18] Teixeira AL, Colpo GD, Fries GR, Bauer IE, Selvaraj S (2019). Biomarkers for bipolar disorder: current status and challenges ahead. Expert Rev Neurother.

[19] Kishi T, Yoshimura R, Ikuta T, Iwata N (2018). Brain-derived neurotrophic factor and major depressive disorder: evidence from meta-analyses. Front Psychiatry.

[20] Kautzky A, James GM, Philippe C, Baldinger-Melich P, Kraus C, Kranz GS (2019). Epistasis of HTR1A and BDNF risk genes alters cortical 5-HT1A receptor binding: PET results link genotype to molecular phenotype in depression. Transl Psychiatry.

[21] Aldoghachi AF, Tor YS, Redzun SZ, Lokman KA, Bin, Razaq NAA, Shahbudin AF (2019). Screening of brain-derived neurotrophic factor (BDNF) single nucleotide polymorphisms and plasma BDNF levels among Malaysian major depressive disorder patients. PLoS One.

[22] Fang Y, Qiu Q, Zhang S, Sun L, Li G, Xiao S (2018). Changes in miRNA-132 and miR-124 levels in non-treated and citalopram-treated patients with depression. J Affect Disord.

[23] El Fatimy R, Li S, Chen Z, Mushannen T, Gongala S, Wei Z (2018). MicroRNA-132 provides neuroprotection for tauopathies via multiple signaling pathways. Acta Neuropathol.

[24] Jia Y, Liu L, Sheng C, Cheng Z, Cui L, Li M (2019). Increased serum levels of cortisol and inflammatory cytokines in people with depression. J Nerv Ment Dis.

[25] Yuan H, Mischoulon D, Fava M, Otto MW (2018). Circulating microRNAs as biomarkers for depression: many candidates, few finalists. J Affect Disord.

[26] Rasheed M, Asghar R, Firdoos S, Ahmad N, Nazir A, Ullah KM (2022). A systematic review of circulatory microRNAs in major depressive disorder: potential biomarkers for disease prognosis. Int J Mol Sci.

[27] Chen J, Bai S-J, Li W, Zhou C, Zheng P, Fang L (2018). Urinary biomarker panel for diagnosing patients with depression and anxiety disorders. Transl Psychiatry.

[28] Chan MK, Cooper JD, Bot M, Steiner J, Penninx BWJH, Bahn S (2016). Identification of an immune-neuroendocrine biomarker panel for detection of depression: a joint effects statistical approach. Neuroendocrinology.

[29] Horne R, Foster JA (2018). Metabolic and microbiota measures as peripheral biomarkers in major depressive disorder. Front Psychiatry.

[30] Han SYS, Cooper JD, Ozcan S, Rustogi N, Penninx BWJH, Bahn S (2019). Integrating proteomic, sociodemographic and clinical data to predict future depression diagnosis in subthreshold symptomatic individuals. Transl Psychiatry.

[31] Galvão AC, de M, Almeida RN, de Sousa Júnior GM, Leocadio-Miguel MA, Palhano-Fontes F (2021). Potential biomarkers of major depression diagnosis and chronicity. PLoS One.

[32] Lee Y, Kang B-H, Kang M, Chung DR, Yi G-S, Lee LP (2020). Nanoplasmonic on-chip PCR for rapid precision molecular diagnostics. ACS Appl Mater Interfaces.

[33] González-González E, Mendoza-Ramos JL, Pedroza SC, Cuellar-Monterrubio AA, Márquez-Ipiña AR, Lira-Serhan D (2019). Validation of use of the miniPCR thermocycler for Ebola and Zika virus detection. PLoS One.

[34] Lan L, Xu D, Ye G, Xia C, Wang S, Li Y (2020). Positive RT-PCR test results in patients recovered from COVID-19. JAMA.

[35] Crutchfield CA, Thomas SN, Sokoll LJ, Chan DW (2016). Advances in mass spectrometry-based clinical biomarker discovery. Clin Proteom.

[36] Gadad BS, Jha MK, Czysz A, Furman JL, Mayes TL, Emslie MP (2018). Peripheral biomarkers of major depression and antidepressant treatment response: current knowledge and future outlooks. J Affect Disord.

[37] Cross TG, Hornshaw MP (2016). Can LC and LC-MS ever replace immunoassays?. J Appl Bioanal.

[38] McShane AJ, Bunch DR, Wang S (2016). Therapeutic drug monitoring of immunosuppressants by liquid chromatography–mass spectrometry. Clin Chim Acta.

[39] Florkowski C, Don-Wauchope A, Gimenez N, Rodriguez-Capote K, Wils J, Zemlin A (2017). Point-of-care testing (POCT) and evidence-based laboratory medicine (EBLM) – does it leverage any advantage in clinical decision making?. Crit Rev Clin Lab Sci.

[40] Yáñez-Sedeño P, Campuzano S, Pingarrón J (2017). Multiplexed electrochemical immunosensors for clinical biomarkers. Sensors.

[41] Pakchin PS, Nakhjavani SA, Saber R, Ghanbari H, Omidi Y (2017). Recent advances in simultaneous electrochemical multi-analyte sensing platforms. TrAC Trends Anal Chem.

[42] Baraket A, Lee M, Zine N, Sigaud M, Bausells J, Errachid A (2017). A fully integrated electrochemical biosensor platform fabrication process for cytokines detection. Biosens Bioelectron.

[43] Tanak AS, Muthukumar S, Krishnan S, Schully KL, Clark DV, Prasad S (2021). Multiplexed cytokine detection using electrochemical point-of-care sensing device towards rapid sepsis endotyping. Biosens Bioelectron.

[44] Zhang W, Wang R, Luo F, Wang P, Lin Z (2020). Miniaturized electrochemical sensors and their point-of-care applications. Chin Chem Lett.

[45] Morales MA, Halpern JM (2018). Guide to selecting a biorecognition element for biosensors. Bioconjug Chem.

[46] Labib M, Sargent EH, Kelley SO (2016). Electrochemical methods for the analysis of clinically relevant biomolecules. Chem Rev.

[47] Wilson A (2018). Application of electronic-nose technologies and VOC-biomarkers for the noninvasive early diagnosis of gastrointestinal diseases. Sensors.

[48] Panahi Z, Custer L, Halpern JM (2021). Recent advances in non-enzymatic electrochemical detection of hydrophobic metabolites in biofluids. Sens Actuators Rep.

[49] Panahi Z, Merrill MA, Halpern JM (2020). Reusable cyclodextrin-based electrochemical platform for detection of trans-resveratrol. ACS Appl Polym Mater.

[50] Oh C, Park B, Li C, Maldarelli C, Schaefer JL, Datta-Chaudhuri T (2021). Electrochemical immunosensing of interleukin-6 in human cerebrospinal fluid and human serum as an early biomarker for traumatic brain injury. ACS Meas Sci Au.

[51] Punj S, Sidhu D, Bhattacharya D, Wang M, Wong PK (2020). An electrochemical biosensor platform for rapid immunoanalysis of physiological fluids. IEEE Open J Nanotechnol.

[52] Tugce Yaman Y, Akbal Vural O, Bolat G, Abaci S (2022). Peptide nanotubes/self-assembled polydopamine molecularly imprinted biochip for the impedimetric detection of human Interleukin-6. Bioelectrochemistry.

[53] Wang Z, Yang S, Wang Y, Feng W, Li B, Jiao J (2020). A novel oriented immunosensor based on AuNPs-thionine-CMWCNTs and staphylococcal protein A for interleukin-6 analysis in complicated biological samples. Anal Chim Acta.

[54] Chen H, Choo TK, Huang J, Wang Y, Liu Y, Platt M (2016). Label-free electronic detection of interleukin-6 using horizontally aligned carbon nanotubes. Mater Des.

[55] Pruna R, Palacio F, Baraket A, Zine N, Streklas A, Bausells J (2018). A low-cost and miniaturized potentiostat for sensing of biomolecular species such as TNF-α by electrochemical impedance spectroscopy. Biosens Bioelectron.

[56] Filik H, Avan AA (2020). Electrochemical immunosensors for the detection of cytokine tumor necrosis factor alpha: a review. Talanta.

[57] Jampasa S, Siangproh W, Laocharoensuk R, Vilaivan T, Chailapakul O (2018). Electrochemical detection of C-reactive protein based on anthraquinone-labeled antibody using a screen-printed graphene electrode. Talanta.

[58] Zhang J, Zhang W, Guo J, Wang J, Zhang Y (2017). Electrochemical detection of C-reactive protein using copper nanoparticles and hybridization chain reaction amplifying signal. Anal Biochem.

[59] Xu H, Luo J, Wang Y, Song Y, Wang L, Cai X. Label-free electrochemical detection of brain-derived neurotrophic factor based on a novel immune microelectrode array. 2017 IEEE 17th Int Conf Nanotechnol. 2017:584–9.

[60] Bockaj M, Fung B, Tsoulis M, Foster WG, Soleymani L (2018). Method for electrochemical detection of brain derived neurotrophic factor (BDNF) in plasma. Anal Chem.

[61] Chahin N, Uribe LA, Debela AM, Thorimbert S, Hasenknopf B, Ortiz M (2018). Electrochemical primer extension based on polyoxometalate electroactive labels for multiplexed detection of single nucleotide polymorphisms. Biosens Bioelectron.

[62] Malpartida-Cardenas K, Miscourides N, Rodriguez-Manzano J, Yu L-S, Moser N, Baum J (2019). Quantitative and rapid Plasmodium falciparum malaria diagnosis and artemisinin-resistance detection using a CMOS Lab-on-Chip platform. Biosens Bioelectron.

[63] Stojanovic Z, Erdőssy J, Keltai K, Scheller FW, Gyurcsányi RE (2017). Electrosynthesized molecularly imprinted polyscopoletin nanofilms for human serum albumin detection. Anal Chim Acta.

[64] Kaur G, Tomar M, Gupta V (2016). Realization of a label-free electrochemical immunosensor for detection of low density lipoprotein using NiO thin film. Biosens Bioelectron.

[65] Rodriguez-Silva AA, Movil-Cabrera O, Oliveira dos Anjos CT, Staser JA (2016). Supercapacitor-based biosensor for low density lipoprotein detection. J Electrochem Soc.

[66] Arral ML, Halpern JM (2018). Electrochemical detection of N^G^-hydroxy-L-arginine. ECS Trans.

[67] Arral ML, Tooley C, Ziino E, Halpern JM (2020). Elucidating the electrochemical mechanism of N^G^-hydroxy-L-arginine. J Electrochem Soc.

[68] Singh R, Kashyap S, Kumar S, Abraham S, Gupta TK, Kayastha AM (2017). Excellent storage stability and sensitive detection of neurotoxin quinolinic acid. Biosens Bioelectron.

[69] Brooks EL, Mutengwa VS, Abdalla A, Yeoman MS, Patel BA (2019). Determination of tryptophan metabolism from biological tissues and fluids using high performance liquid chromatography with simultaneous dual electrochemical detection. Analyst.

[70] Sadok I, Tyszczuk-Rotko K, Mroczka R, Staniszewska M (2020). Simultaneous voltammetric analysis of tryptophan and kynurenine in culture medium from human cancer cells. Talanta.

[71] Hu M, Fritsch I (2016). Application of electrochemical redox cycling: toward differentiation of dopamine and norepinephrine. Anal Chem.

[72] Manbohi A, Ahmadi SH (2019). Sensitive and selective detection of dopamine using electrochemical microfluidic paper-based analytical nanosensor. Sens Bio-Sens Res.

[73] Ostertag BJ, Cryan MT, Serrano JM, Liu G, Ross AE (2022). Porous carbon nanofiber-modified carbon fiber microelectrodes for dopamine detection. ACS Appl Nano Mater.

[74] Weese-Myers ME, Ross AE (2021). Characterization of electroactive amino acids with fast-scan cyclic voltammetry. J Electrochem Soc.

[75] Li Y, Jarosova R, Weese-Myers ME, Ross AE (2022). Graphene-fiber microelectrodes for ultrasensitive neurochemical detection. Anal Chem.

[76] Li W, Qian D, Li Y, Bao N, Gu H, Yu C (2016). Fully-drawn pencil-on-paper sensors for electroanalysis of dopamine. J Electroanal Chem.

[77] Iwai NT, Kramaric M, Crabbe D, Wei Y, Chen R, Shen M (2018). GABA detection with nano-ITIES pipet electrode: a new mechanism, water/DCE–octanoic acid interface. Anal Chem.

[78] Li Y, He R, Niu Y, Li F (2019). Paper-based electrochemical biosensors for point-of-care testing of neurotransmitters. J Anal Test.

[79] Rahman R, Mini P, Menamparambath M (2021). Transition metal oxide based non-enzymatic electrochemical sensors: an arising approach for the meticulous detection of neurotransmitter biomarkers. Electrochem Sci Adv.

[80] Moulaee K, Neri G (2021). Electrochemical amino acid sensing: a review on challenges and achievements. Biosens 2021.

[81] Matsunaga T, Kondo T, Shitanda I, Hoshi Y, Itagaki M, Tojo T (2021). Sensitive electrochemical detection of l-Cysteine at a screen-printed diamond electrode. Carbon N Y.

[82] Tooley C, Gasperoni C, Marnoto S, Halpern J (2018). Evaluation of metal oxide surface catalysts for the electrochemical activation of amino acids. Sensors.

[83] Jirjees Dhulkefl A, Atacan K, Bas SZ, Ozmen M (2020). An Ag–TiO 2–reduced graphene oxide hybrid film for electrochemical detection of 8-hydroxy-2′-deoxyguanosine as an oxidative DNA damage biomarker. Anal Methods.

[84] Martins GV, Tavares APM, Fortunato E, Sales MGF (2017). Paper-based sensing device for electrochemical detection of oxidative stress biomarker 8-hydroxy-2′-deoxyguanosine (8-OHdG) in point-of-care. Sci Rep.

[85] Ren T, Bramlitt SE, LaFreniere JMJ, Seitz WR, Halpern JM (2021). Conformation-based stimuli-response sensors: Strategies for optimizing electrochemical and FRET transduction. Sens Actuators Rep.

[86] Wang J, Lu Z, Tang H, Wu L, Wang Z, Wu M (2017). Multiplexed electrochemical detection of MiRNAs from sera of glioma patients at different stages via the novel conjugates of conducting magnetic microbeads and diblock oligonucleotide-modified gold nanoparticles. Anal Chem.

[87] Azimzadeh M, Rahaie M, Nasirizadeh N, Ashtari K, Naderi-Manesh H (2016). An electrochemical nanobiosensor for plasma miRNA-155, based on graphene oxide and gold nanorod, for early detection of breast cancer. Biosens Bioelectron.

[88] Robertson NM, Toscano AE, LaMantia VE, Hizir MS, Rana M, Balcioglu M (2017). Unlocked nucleic acids for miRNA detection using two dimensional nano-graphene oxide. Biosens Bioelectron.

[89] Chang Y, Xu S, Li Y, Hu W, Li H, Yuan R (2021). DNA three-way junction with multiple recognition regions mediated an unconfined DNA walker for electrochemical ultrasensitive detection of miRNA-182-5p. Anal Chem.

[90] Liang D, Zhang X, Wang Y, Huo T, Qian M, Xie Y (2022). Magnetic covalent organic framework nanospheres-based miRNA biosensor for sensitive glioma detection. Bioact Mater.

[91] Sánchez-Tirado E, González-Cortés A, Yudasaka M, Iijima S, Langa F, Yáñez-Sedeño P (2017). Electrochemical immunosensor for the determination of 8-isoprostane aging biomarker using carbon nanohorns-modified disposable electrodes. J Electroanal Chem.

[92] Hasanzadeh M, Mokhtari F, Shadjou N, Eftekhari A, Mokhtarzadeh A, Jouyban-Gharamaleki V (2017). Poly arginine-graphene quantum dots as a biocompatible and non-toxic nanocomposite: layer-by-layer electrochemical preparation, characterization and non-invasive malondialdehyde sensory application in exhaled breath condensate. Mater Sci Eng C.

[93] Jafari M, Solhi E, Tagi S, Hasanzadeh M, Jouyban-Gharamaleki V, Jouyban A (2019). Non-invasive quantification of malondialdehyde biomarker in human exhaled breath condensate using self-assembled organic-inorganic nanohybrid: a new platform for early diagnosis of lung disease. J Pharm Biomed Anal.

[94] Zamani-Kalajahi M, Hasanzadeh M, Shadjou N, Khoubnasabjafari M, Ansarin K, Jouyban-Gharamaleki V (2015). Electrodeposition of taurine on gold surface and electro-oxidation of malondialdehyde. Surf Eng.

[95] Kordasht HK, Hasanzadeh M, Seidi F, Alizadeh PM (2021). Poly (amino acids) towards sensing: Recent progress and challenges. TrAC Trends Anal Chem.

[96] Sinha A, Dhanjai, Jain R, Zhao H, Karolia P, Jadon N (2018). Voltammetric sensing based on the use of advanced carbonaceous nanomaterials: a review. Microchim Acta.

[97] Tang CK, Vaze A, Shen M, Rusling JF (2016). High-throughput electrochemical microfluidic immunoarray for multiplexed detection of cancer biomarker proteins. ACS Sens.

[98] Feeney SG, LaFreniere JMJ, Halpern JM (2021). Perspective on nanofiber electrochemical sensors: design of relative selectivity experiments. Polymers (Basel).

[99] Torrente-Rodríguez RM, Campuzano S, Ruiz-Valdepeñas Montiel V, Gamella M, Pingarrón JM (2016). Electrochemical bioplatforms for the simultaneous determination of interleukin (IL)-8 mRNA and IL-8 protein oral cancer biomarkers in raw saliva. Biosens Bioelectron.

[100] Hirose A, Terauchi M, Akiyoshi M, Owa Y, Kato K, Kubota T (2016). Depressive symptoms are associated with oxidative stress in middle-aged women: a cross-sectional study. Biopsychosoc Med.

[101] Noushin T, Tabassum S. Multiplexed electrochemical sensor for real-time monitoring of inflammatory biomarkers. 2021 IEEE Sensors. 2021;1–4.

